# Prevalence and risk factors of low birth weight in a rural district of Bangladesh: a prospective cohort study

**DOI:** 10.1186/s12887-026-06960-x

**Published:** 2026-05-12

**Authors:** Pallab Bhattacharjee, Md Shafiqul Islam, Arunangshu Roy, Zannatul Ferdush Amin, Nayema Bintye Rahman, M A Shahed, Fahmeda Akter, Nabidul Haque Chowdhury, Dipak Kumar Mitra, Rasheda Khanam, Abdullah H. Baqui, Salahuddin Ahmed

**Affiliations:** 1 Projahnmo Research Foundation, Dhaka, Bangladesh; 2 Department of International Health, Johns Hopkins Bloomberg School of Public Health, Baltimore, MD United States of America; 3 Department of Public Health, School of Health and Life Sciences, North South University, Dhaka, Bangladesh

**Keywords:** Low birthweight, Risk factors, Sylhet, Bangladesh, Incidence

## Abstract

**Background:**

Low birth weight (LBW, < 2500 gm) is a major contributor to infant morbidity and mortality and is strongly linked to childhood developmental delays.

**Methods:**

To estimate the prevalence of LBW and identify its risk factors, we analyzed data from a community-based prospective cohort study titled, “Aetiology of Neonatal Infections in South Asia”. In Bangladesh, the study was conducted in two sub-districts of Sylhet district. The study followed 24,271 pregnant women from 2011 to 2013. The final analysis included 17,643 singleton live-born babies with available birthweight data. To identify risk factors of LBW, we calculated adjusted risk ratios (aRRs) and 95% confidence intervals (CIs) using multivariable modified Poisson regression with robust variance.

**Results:**

The prevalence of LBW was 27.61% (95% CI: 26.96–28.28). Maternal factors included undernutrition (MUAC < 22 cm) [aRR 1.19 (95% CI:1.13–1.25)], tobacco use [aRR 1.16 (95% CI: 1.09–1.23)], no antenatal visits with skilled providers [aRR 1.16 (95% CI: 1.07–1.25)], lack of tetanus toxoid vaccination [aRR 1.17 (95% CI:1.11–1.25)], absence of antenatal iron use [aRR 1.10 (95% CI:1.02–1.19)], and pregnancy complications [aRR 1.12 (95% CI:1.05–1.19)]. Other significant factors were lowest household quintile [aRR 1.45 (95% CI: 1.32–1.59)], female sex and residence ≥ 15 km from sub-district hospitals.

**Conclusions:**

The burden of LBW was high in rural Bangladesh. Multiple modifiable maternal factors, including undernutrition, tobacco use, lack of tetanus toxoid vaccination, absence of antenatal iron use and no antenatal care, were associated with increased risk of LBW. Maternal and child health programs should prioritize interventions to address these factors.

**Supplementary Information:**

The online version contains supplementary material available at 10.1186/s12887-026-06960-x.

## Introduction

The World Health Organization (WHO) defines low birth weight (LBW) as the weight of a live-born infant less than 2500 g at birth, regardless of gestational age [1]. In 2015, 20.5 million (14.6%) babies were born with LBW worldwide, 91% of which occurred in low- and middle-income countries (LIMCs). South Asia (48%) and sub-Saharan Africa (24%) had the highest burden [2]. Low birth weight (LBW) primarily results from two etiological factors: preterm birth, defined as delivery before 37 completed weeks of gestation, and intrauterine growth restriction (IUGR), a condition marked by suboptimal fetal growth in utero [3–6]. LBW remains a major public health problem globally that is associated with multiple short-term and long-term adverse outcomes [7]. LBW babies are at significantly higher risk of morbidity and mortality and other physical and neurodevelopmental impairments [8]. The risk of mortality increases greatly as birthweight decreases and the risk of neonatal mortality in very LBW infants (< 1500 gram) is about 10 times higher compared to normal birthweight babies [9].

**Fig. 1:**
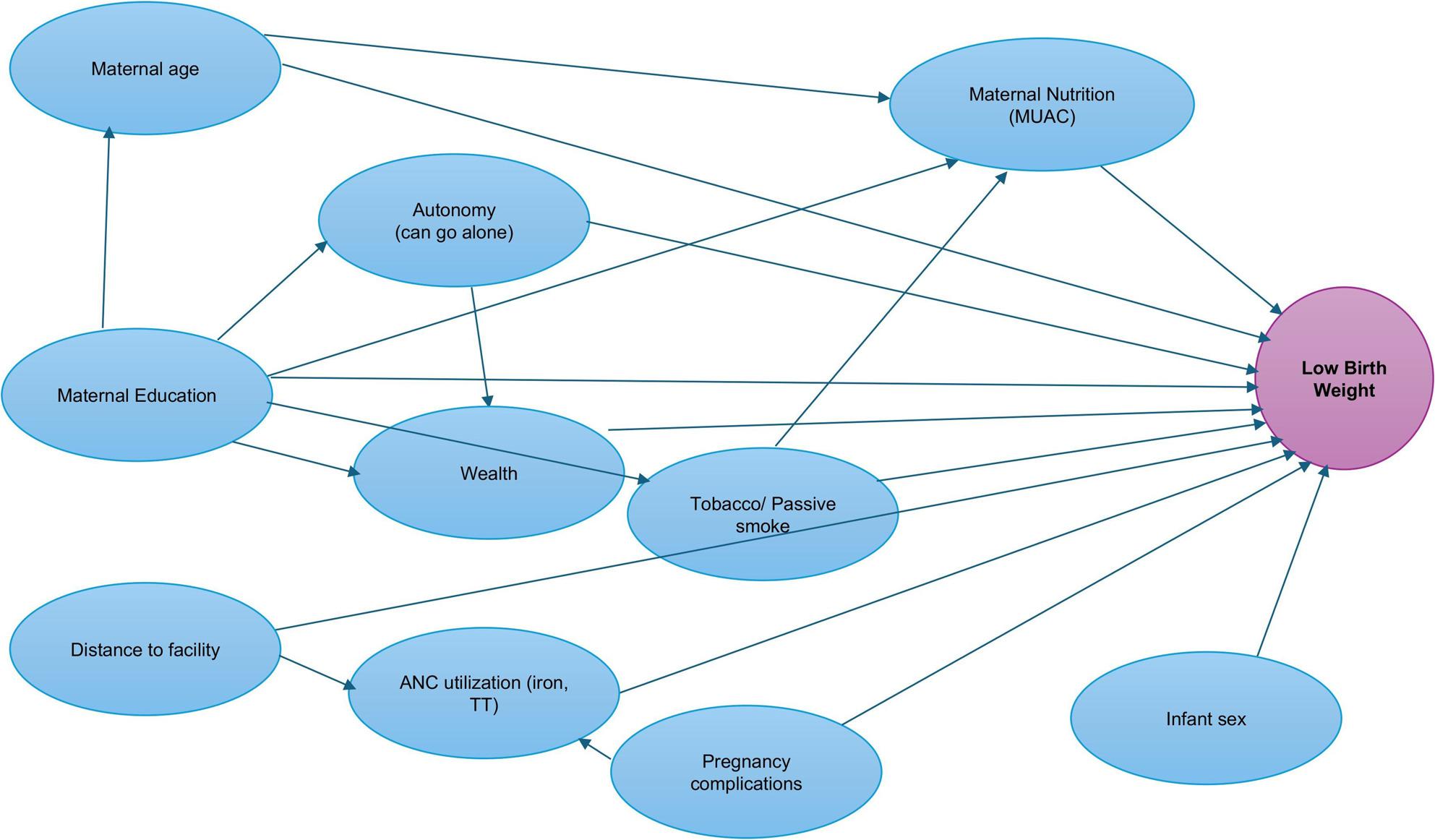
Directed acyclic graph (DAG) to identify minimal sufficient adjustment set of independent variables for low birth weight. Based on DAG model was as Fig. 1 as follows: maternal age was adjusted by maternal education and household wealth; maternal nutrition was adjusted by maternal age, maternal education, household wealth and maternal smoking habit; maternal smoking habit was adjusted by maternal education and maternal age; household wealth was adjusted by maternal age and maternal education; ANC utilization was adjusted by maternal education, household wealth, maternal autonomy, and distance to facility; maternal autonomy was adjusted by maternal education and household wealth; distance to facility was adjusted by household wealth; infant sex was adjusted by none

The World Health Organization (WHO) set a global nutrition target of a 30% reduction in low birth weight between 2012 and 2025; however, due to slow progress globally, the World Health Assembly has extended this target to 2030 to accelerate efforts to reduce the prevalence of low birth weight [10]. The rate of low birth weight in Bangladesh was 23% according to the National LBW Survey 2016, with a target to reduce it to 16% by 2025. As of the MICS 2019 survey, the rate had declined to 14.8%, indicating progress toward achieving the target [11]. Despite a substantial decline in the LBW prevalence between 2011 and 2019, recent data indicate that Bangladesh has achieved substantial progress in reducing the prevalence of low birth weight, aligning more closely with the averages reported in many low- and middle-income countries [12]. Extensive research has sought to identify the determinants of LBW [13–15]. Earlier findings from Bangladesh and elsewhere suggest that birth weight in Bangladesh is impacted by a variety of factors, including socio-demographic characteristics, maternal characteristics, and health care related and biological factors [14, 16, 17]. To select the potential risk factors of LBW, we listed all the exposure variables available in the data set. Based on a comprehensive literature review, a directed acyclic graph (DAG) was constructed to identify a minimum set of confounding variables for adjustment (Fig. 1) [18]. A few established risk factors of LBW, namely genetics and maternal infections during pregnancy were not measured in the parent study.

Most of the past studies were cross-sectional, and birth weights were often assessed by the mother’s recall, resulting in measurement errors and incorrect estimates of LBW prevalence [19, 20]. The present study estimated the prevalence of LBW and identified the antenatal, intrapartum and neonatal risk factors using data from a well-designed cohort study with real-time and valid measures of birth weight and risk factors data prospectively collected during pregnancy.

## Methods

### Study design

This study used prospectively collected data from a community-based cohort study, Aetiology of Neonatal Infections in South Asia (ANISA). ANISA was a multi-country study conducted in Bangladesh, India, and Pakistan. The study aimed to determine the burden of bacterial and viral infections in newborns and young infants. The details of the study methods were published elsewhere [21, 22]. This analysis uses data from the Bangladesh site only.

### Study settings and population

This prospective, community-based study was conducted by the Project for Advancing Health of Newborn and Mothers (Projahnmo) study group between June 2011 and December 2013 in two sub-districts, Kanaighat and Zakiganj of Sylhet district in Bangladesh located at the north-east part of the country. The study area had a population of about 400,000 with an approximate annual birth of 9,000 [21–24]. The population of each sub-district is served by a 50-bed hospital providing both out-patient and in-patient care. The study area represents a typical rural community; most of the population are engaged in agricultural work. The area was previously mapped, all households were enumerated, and household sociodemographic and geographic information system (GIS) data for households, health workers’ residences and health facilities in the community were collected.

### Study procedures

The community health workers (CHWs) with at least 10th grade education were locally recruited and trained by the project staff. CHWs maintained lists of all married women of reproductive age (MWRA) in their assigned areas. They made two-monthly home visits to update the MWRA list and identify pregnant women based on reported last menstrual period (LMP). During the study period, the CHWs identified a total of 28,960 pregnant women, invited them to participate in the study, and enrolled after explaining the study procedures and obtaining informed consent. The consent included future utilization of data collected. CHWs provided a basic package of maternal and newborn health (MNH) care to all women in the study area, including counseling and education on preventive care, recognition of and care-seeking for maternal and newborn danger signs, and referral for emergency care during antepartum, intrapartum and postpartum periods.

In the parent study, the CHWs followed the pregnant women twice in the antenatal period and ten times in the postpartum period up to 60 days to collect the study-related data and to provide the maternal and newborn care [21]. For this analysis we used data collected on the antenatal visits and the first postnatal visit only. They conducted the first antenatal visit immediately after confirming pregnancy and collected data on women’s demographic and socioeconomic characteristics, as well as data on their ability to decide to go to the health center alone. A second visit was made during the 29th week of pregnancy to provide information related to maternal and newborn care. The first postpartum visit was made as soon as possible after birth but no later than 7 days of delivery and data were collected on antenatal care, delivery characteristics, newborn’s vital status and weight, place of delivery, birth attendants, and self-reported antepartum and intrapartum complications. Data were collected at home visits using paper-based case record forms. Data were entered using a custom-made data entry system with built-in range and consistency checks.

### Assessment of variables

The primary outcome of the study is the birthweight of the live born infants measured by CHWs on the first postnatal visits at home using standard pediatric scale (Tanita BD-585; precision 10 g). Low birth weight (LBW) was defined as birthweight < 2500 g, in accordance with World Health Organization criteria. In this study, infants with birthweight measured with 72 h are included. Data on exposure variables were collected by CHWs during antenatal visits and the first postnatal visits using a set of questionnaires and assessment tools. Information related to socio-demographic and economic factors (age, education, basic housing materials, sanitation and drinking water source, household belongings, religion, family size, distance from nearest health facility, empowered to go to health facility alone) and maternal MUAC were collected in the first antenatal visits. Data on antenatal care, antenatal complications (high grade fever, excessive bleeding, convulsion, swelling of face or feet, and foul smelling discharge), consumption of iron tablets, Tetanus Toxoid (TT) immunization, gestational age (based on reported LMP and date of delivery), sex of infants, birth interval (interval between previous delivery and index delivery dates) consumption of tobacco during pregnancy, household works during pregnancy were collected on the first postnatal visits. Most of the data were collected based on maternal self-reports. Pregnancy complications were also self-reported, but with prompts for specific symptoms including high fever, excessive bleeding, convulsions, swelling of the face or feet, and foul-smelling discharge. Mid-arm circumferences were measured by the CHWs at the first antenatal visits (12–16 weeks of gestation) using a Teaching Aids at Low Cost (TALC) insertion tape (precision: 1 mm) [25].

### Training and quality control measures

Detailed training and quality assurance procedures have been previously described in the published ANISA protocol [22] Briefly, community health workers (CHWs) received a two-weeks training on the study procedures using a standardized manual and conducted by trained physicians. All CHWs were standardized on birth weight measurements. The weighing scales were calibrated each morning, and calibration records were maintained in a logbook. Clinical training for CHWs and field supervisors took place in the pediatric and obstetric wards of a nearby teaching hospital. Field implementation was supported by structured tools, including a pregnancy register and CHW monthly planner. To maintain data quality and protocol adherence, six-monthly refreshers training was conducted for all field staff, and on-the-job training is imparted regularly to CHWs by supervisory staff. Supervisory visits were conducted routinely, and all data entry was done by trained data clerks using an in-house data entry system with built-in checks on range, consistency, and valid values.

### Statistical analysis

Singleton liveborn infants with data on gestational age and birthweight were included in the analysis. We estimated the prevalence of LBW by calculating the proportion of infants with LBW among all singletons live births with a 95% confidence interval. The association between LBW and potential risk factor variables was determined using a chi-square test of independence or Fisher’s exact test as appropriate. The magnitude of association between LBW and risk factors was examined using a modified robust Poisson regression model for binary data to directly estimate the Risk Ratio and 95% Confidence Interval [26]. We used this method instead of commonly used logistic regression as the latter overestimates the association, especially when the prevalence of the outcome is high [27]. Independent variables were selected based on the DAG (Figure-1) that included maternal age in year, maternal education, distance to nearest facility, maternal autonomy, household wealth, ANC utilization, maternal TT receipt, maternal tobacco consumption, maternal passive smoking, pregnancy complications, maternal nutrition (MUAC) and infant sex. We created a household wealth index using principal component analysis (PCA) with data on household assets, type of housing materials, type of latrines, source of drinking water, and household possessions, a methodology generally used in Demographic and Health surveys [28]. The first principal component was retained to derive the wealth index. The first component had an eigenvalue of 5.28 and explained 15.1% of the total variance. The households were classified into five groups based on the wealth index score (Lowest quintile or Poorest, second lowest quintile, Middle quintile, second highest quintile, Highest quintile or Richest). Only two independent variables- MUAC and number of antenatal care visits had 6.4% and 1.4% missing values respectively and missing values were imputed using hot-deck method [29]. The imputation method was chosen based on its suitability for our data [30]. We conducted both bivariate and multivariable regression models. We included all variables examined in the bivariate analysis in the multivariable model, as all showed a strong association with the outcome. Model adequacy was assessed using deviance and Pearson goodness-of-fit statistics. The goodness-of-fit tests did not indicate lack of fit (*p* > 0.05). Dispersion was evaluated using the Pearson chi-square statistic divided by its degrees of freedom, which was 0.72, indicating no evidence of overdispersion. Robust standard errors were used to account for potential deviations from the Poisson variance assumption. Multicollinearity was assessed using Generalized Variance Inflation Factors (GVIFs). All adjusted GVIF values were below two, indicating no concerning multicollinearity among covariates.

All analyses were done using Stata 18 (StataCorp. 2023. Stata Statistical Software: Release 18. College Station, TX: Stata Corp LLC.) and R statistical software. P value < 0.05 was considered statistically significant.

## Results

**Fig. 2:**
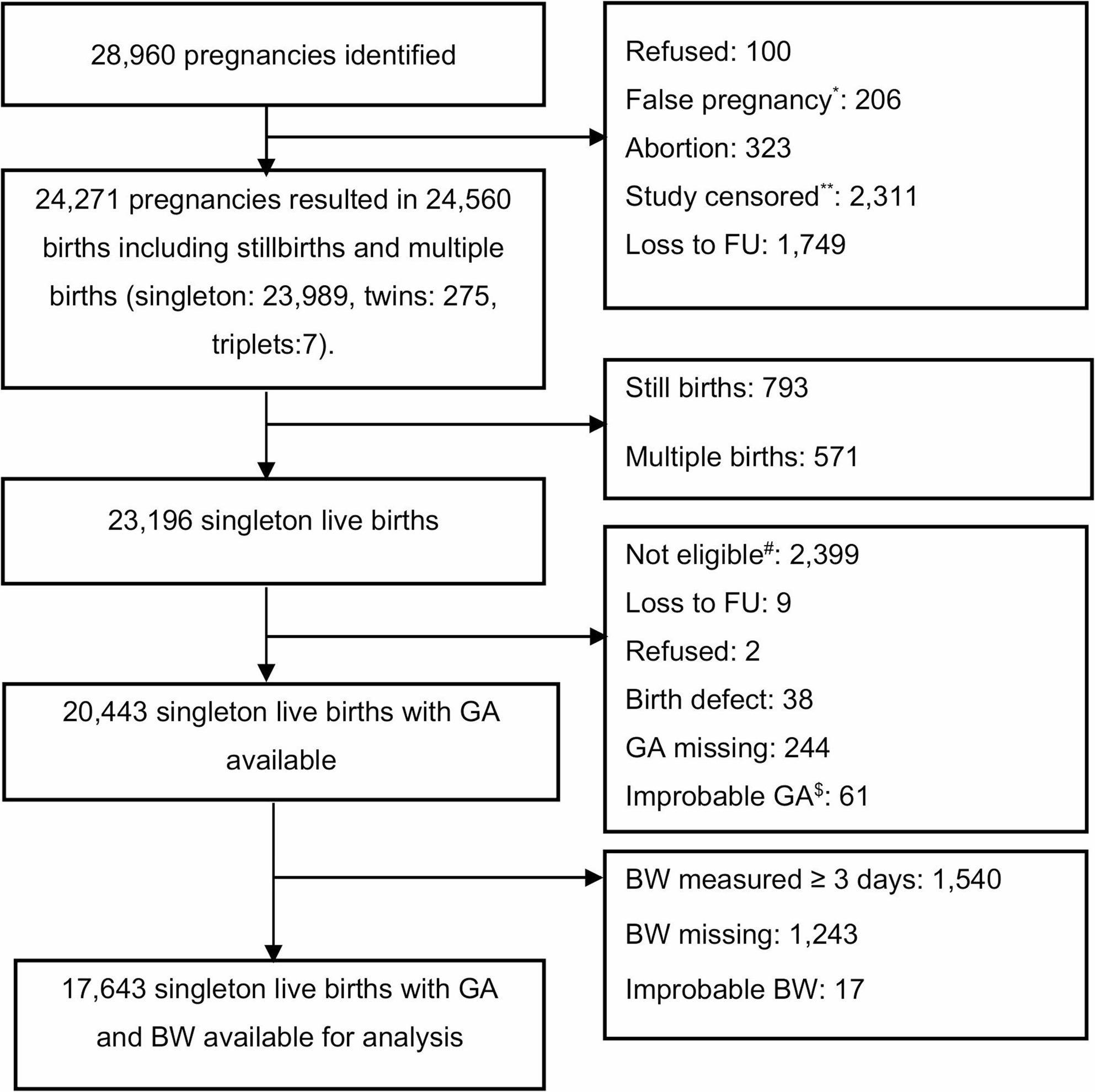
Study Profile. *False Pregnancy: The initial detection of pregnancy based on reported amenorrhea was due to other reasons than pregnancy. **Study censored: Pregnant women did not have pregnancy outcome as of the study ended administratively. #Not eligible: As per the primary study eligibility, the birth-conditioned baby was identified after 7 days of childbirth. $Improbable birth weight: BW recorded extremely high which were identified later in the database and could not be validated in the field

During the study period, 28,960 pregnant women were identified, of whom 100 refused participations, 206 were identified as false pregnancies, 323 had miscarriages and 2,311 did not have a pregnancy outcome before the study ended (censored). Out of 24,271 deliveries, there were 24,560 childbirths. Among all childbirths, 23,196 were live birth singleton and initially included in the analysis, while 550 were twins, 21 were triplets, and 793 were stillbirths who were excluded. Out of all the singleton live births, 2,399 were not eligible for enrollment in the parent study, because required that infants were met within 7 days of birth; 2,783 had missing birthweights, 9 were lost to follow up, 2 refused participation, 38 had birth defects, 244 had missing gestational age (GA), and 61 had improbable GA. Finally, 17,643 singleton newborns were available for analysis (Fig. 2).

The prevalence of LBW in our study population was 27.61% (95% CI: 26.96%-28.28%). Table 1 shows the distribution of maternal and household characteristics. One-third of the women were 20–24 years of age group, while another quarter was 25–29 years old. About 25% of the mothers had no formal schooling, approximately 23% of women had MUAC measurement < 22 cm, and nearly 17% women reported being able to visit a health facility alone. About 16% of women reported using tobacco products (either smoking or chewing tobacco leaf), and nearly 62% reported being exposed to passive smoking during pregnancy. According to the distribution of household wealth quintiles, 20.27% of women were classified in the lowest wealth quintile, while 19.09% were in the highest quintile. All these factors were significantly associated with LBW using the chi square test of independence.

**Table 1:** Association of maternal and household characteristics with low birthweight

Variables	Low BW	Normal BW	Total	*P*-value
	*N* (%) ^a^	*N* (%) ^a^	*N* (%) ^b^	
N	4,872 (27.61)	12,771 (72.39)	17,643 (100)	
Maternal characteristics				
Age, years				
< 20	588 (34.07)	1,138 (65.93)	1,726 (9.78)	< 0.01
20–24	1,781 (30.27)	4,102 (69.73)	5,883 (33.34)	
25–29	1,107 (24.97)	3,326 (75.03)	4,433 (25.13)	
30–34	893 (24.05)	2,820 (75.95)	3,713 (21.05)	
≥35	503 (26.64)	1,385 (73.36)	1,888 (10.7)	
Education				
None	1,303 (30.14)	3,020 (69.86)	4,323 (24.5)	< 0.01
Primary	1,927 (28.92)	4,737 (71.08)	6,664 (37.77)	
Secondary or higher	1,642 (24.67)	5,014 (75.33)	6,656 (37.73)	
Mid-upper arm circumference, cm				
< 22	1,329 (32.89)	2,712 (67.11)	4,041 (22.9)	< 0.01
≥ 22	3,543 (26.05)	10,059 (73.95)	13,602 (77.1)	
Tobacco consumption during pregnancy				
No	3,988 (26.85)	10,863 (73.15)	14,851 (84.18)	< 0.01
Yes	884 (31.66)	1,908 (68.34)	2,792 (15.82)	
Passive smoking during pregnancy				
No	1,714 (25.39)	5,037 (74.61)	6,751 (38.26)	< 0.01
Yes	3,158 (28.99)	7,734 (71.01)	10,892 (61.74)	
Can go to health facility alone				
No	4,150 (28.19)	10,574 (71.81)	14,724 (83.46)	< 0.01
Yes	722 (24.73)	2,197 (75.27)	2,919 (16.54)	
Household characteristics				
Household wealth quintiles				
1 (Lowest)	1,184 (33.11)	2,392 (66.89)	3,576 (20.27)	< 0.01
2	1,164 (31.76)	2,501 (68.24)	3,665 (20.77)	
3	1,024 (27.56)	2,692 (72.44)	3,716 (21.06)	
4	803 (24.2)	2,515 (75.8)	3,318 (18.81)	
5 (Highest)	697 (20.69)	2,671 (79.31)	3,368 (19.09)	

*BW* Birthweight

^a^ Row percentage

^b^ Column percentage

Table 2 shows the distribution of pregnancy characteristics, service factors, and infant factors and their association with LBW. Approximately 14% of respondents reported experiencing any complications during pregnancy. About 40% women did not have any antenatal care visit to a skilled healthcare providers, while 17% received antenatal care (ANC)from skilled healthcare providers for 4 or more times. More than 90% women received iron supplementation during pregnancy. About 78% of women received at least 2 doses of TT immunization in their lifetime. About 40% of the households were located more than 15 km away from the nearest sub-district hospital and half of the infants were female. All these factors were also significantly associated with LBW.

**Table 2:** Association of pregnancy, newborn and health service factors with low birthweight

Variables	Low BW	Normal BW	Total	*P*-value
	*N* (%) ^a^	*N* (%) ^a^	*N* (%) ^b^	
N	4,872 (27.61)	12,771 (72.39)	17,643 (100)	
Pregnancy characteristics				
Antenatal complication during pregnancy				
No	4,154 (27.29)	11,066 (72.71)	15,220 (86.27)	0.02
Yes	718 (29.63)	1,705 (70.37)	2,423 (13.73)	
Service factors				
Antenatal care by a skilled healthcare provider				
No visit	2,137 (30.49)	4,871 (69.51)	7,008 (39.72)	< 0.01
1–3 visits	2,023 (26.78)	5,530 (73.22)	7,553 (42.81)	
≥ 4 visits	712 (23.1)	2,370 (76.9)	3,082 (17.47)	
Taking iron tablet during pregnancy				
No	511 (31.56)	1,108 (68.44)	1,619 (9.18)	< 0.01
Yes	4,361 (27.22)	11,663 (72.78)	16,024 (90.82)	
Lifetime TT-Injection				
No dose	978 (32.49)	2,032 (67.51)	3,010 (17.06)	< 0.01
1 dose	279 (35.01)	518 (64.99)	797 (4.52)	
≥ 2 doses	3,615 (26.13)	10,221 (73.87)	13,836 (78.42)	
Distance to health facility, km				
< 15	2,865 (26.78)	7,834 (73.22)	10,699 (60.64)	< 0.01
≥ 15	2,007 (28.9)	4,937 (71.1)	6,944 (39.36)	
Child factors				
Sex of the infants				
Male	2,249 (25.33)	6,630 (74.67)	8,879 (50.33)	< 0.01
Female	2,623 (29.93)	6,141 (70.07)	8,764 (49.67)	

*BW* Birthweight, *TT* Tetanus toxoid

^a^ Row percentage

^b^ Column percentage

**Fig. 3:**
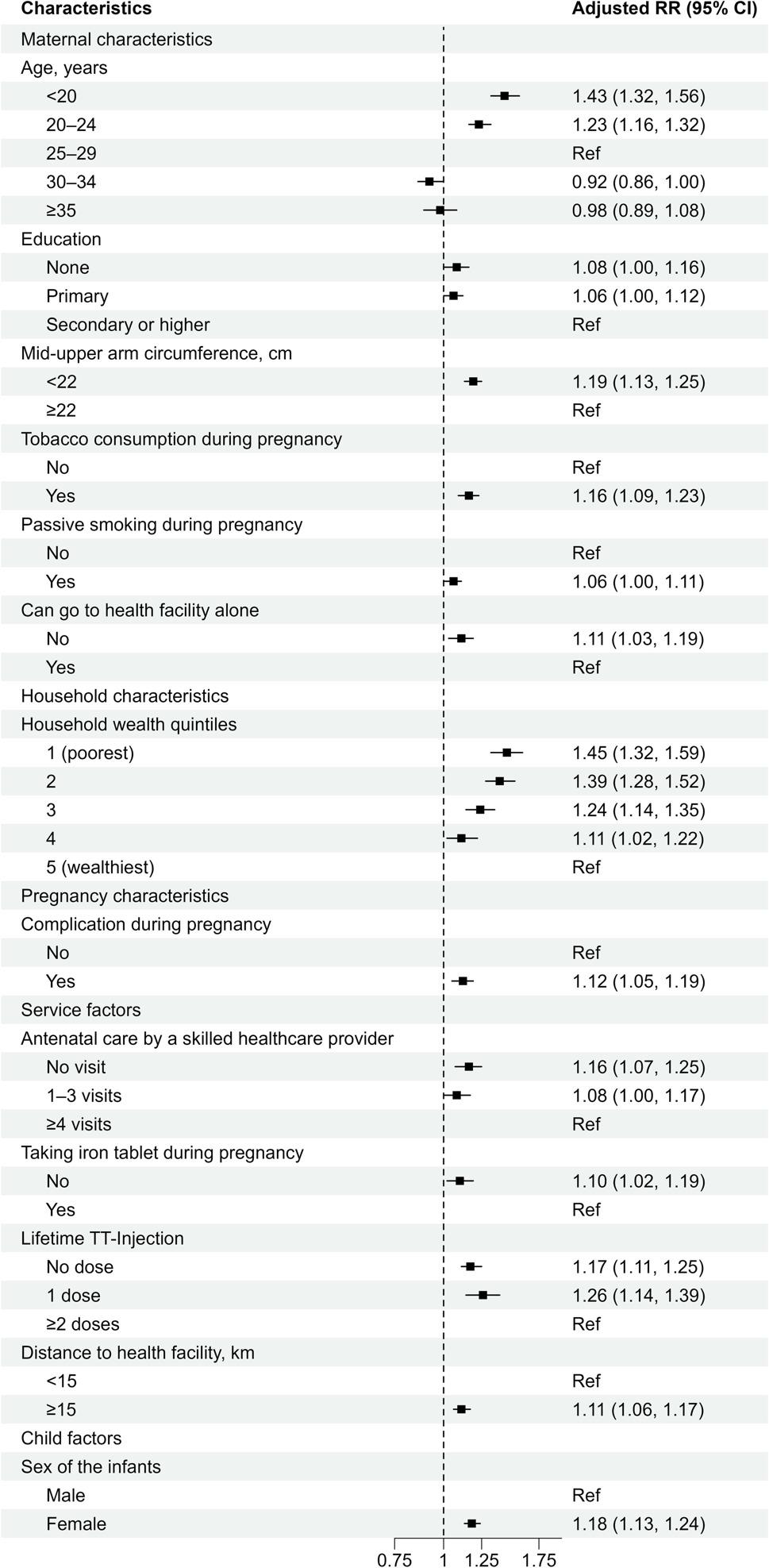
Risk factors of low birthweight: adjusted relative risks and 95% confidence intervals estimated using multivariable modified poisson regression

Figure 3 presents the results of multivariable modified Poisson regression models. In the multivariable model, mother’s lower education level (no education or primary education level), lower household economic status, maternal undernutrition, longer distance of the residence from health facility, antenatal complications, maternal tobacco consumption during pregnancy, no or fewer than 4 ANC visits from skilled providers, no or fewer dose of TT vaccination, inability to go to health facility alone, and female sex of the infants were associated with an increased risk of LBW. In the adjusted model, compared to mothers s’ education level at secondary or above, infants whose mothers with no education had a slightly higher risk of being born LBW [aRR 1.08 (95% CI: 1.00-1.16)]. Infants born into households in the lower wealth quintiles had a significantly higher risk of low birth weight compared with those in the richest quintile. For the lowest, second-lowest, and third-lowest quintiles, the adjusted relative risks (aRR) were 1.45 (95% CI: 1.32–1.59), 1.39 (95% CI: 1.28–1.52), and 1.24 (95% CI: 1.14–1.35), respectively. infants of undernourished mothers with less than 22 cm upper mid-arm circumference (MUAC) had increased risk of LBW [aRR 1.19 (95% CI: 1.13–1.25)] compared to infants born to well-nourished mothers. Higher distance of residence from a health facility (≥ 15 km) was associated with an increased risk of LBW in our study population [aRR 1.11 (95% CI: 1.06–1.17)]. Mothers with antenatal complications during pregnancy had higher risk of delivering LBW infants [aRR 1.12 (95% CI: 1.05–1.19)]. Mothers who consumed tobacco during pregnancy had higher risk of giving birth to LBW infants [aRR 1.16 (95% CI: 1.09–1.23)] compared to mothers who did not consume tobacco during pregnancy. infants born to mothers with no antenatal visits by skilled healthcare providers had higher risk of LBW [aRR 1.16 (95% CI: 1.07–1.25)]. Mothers who did not receive iron supplementation during pregnancy had a higher risk of LBW [aRR 1.10 (95% CI: 1.02–1.19)]. Receiving no dose of antenatal TT immunization increased the risk of LBW infants compared to receiving ≥ 2 doses of TT [aRR 1.17 (95% CI: 1.11–1.25)]. Female infants were at higher risk of being LBW compared to male infants [aRR 1.18 (95% CI: 1.13–1.24)].

## Discussions

In this community-based prospective cohort study in a rural area of the Sylhet district of Bangladesh, we observed a high prevalence of LBW 27.61. A number of maternal, household, and health care–seeking factors were associated with LBW, including, tobacco use, lack of tetanus toxoid vaccination, maternal undernutrition (MUAC < 22 cm), absence of antenatal iron use, pregnancy complications. Other risk factors like low household wealth quintile, and residence ≥ 15 km from sub-district hospitals—were also important contributors to LBW.

The LBW rate we observed was higher than the 20.1% reported in a cross-sectional study from Bangladesh by Islam Pollob et al. (2022) [17]. This difference may partly reflect the declining trend of LBW in Bangladesh over time and partly the limitations of data quality, as Islam Pollob et al. (2022) relied on secondary analysis of the 2017–2018 Bangladesh Demographic and Health Survey (DHS). The DHS obtains birth weight information from health cards or maternal recall, which is prone to misclassification and likely underestimates the true prevalence [31].

The risk factors for low birth weight (LBW) identified in this study are consistent with previous research. Earlier studies have shown significant associations between LBW and socio-economic and structural determinants, particularly maternal education level [32–34]. limited household wealth [15, 35–37], and greater geographic distance from health facilities [16]. These determinants influence key enabling factors for improved maternal nutrition and health care use, which in turn affect birthweight [4, 35, 38].

Better economic conditions are associated with improved health-seeking behaviors among women, including increased utilization of care from qualified healthcare providers. Such access facilitates timely antenatal care (ANC), enabling early diagnosis and management of conditions such as maternal anemia, hypertensive disorders of pregnancy, gestational diabetes, and intrauterine growth restriction, all of which are known contributors to low birthweight (LBW) [39] In this cohort, we observed that mothers who received adequate ANC, defined by the recommended number and timing of visits, had a substantially lower risk of delivering an LBW infant. This finding aligns with evidence from Bangladesh, Ethiopia, and Nepal [15, 40–42]. ANC serves as a critical platform for regular monitoring, early identification and management of maternal and fetal complications, and provision of essential nutritional supplementation, all of which contribute to reducing adverse pregnancy outcomes, including LBW [39, 43].

Our study showed that the nutritional status of women as classified by MUAC was significantly associated with LBW. Infants born to mothers with low MUAC had a 1.2 times higher risk of giving birth to LBW infants compared to infants born to mothers with a MUAC ≥ 22 cm. This study underscores the importance of optimal maternal nutrition during pregnancy for optimal fetal growth, consistent with other studies conducted in Ethiopia and Cambodia [44, 45]. This study showed a significant association between newborn characteristics such as sex and low birth weight. Female neonates had a higher risk of being low birth weight compared to male counterparts. This finding is similar to other studies carried out in Nigeria and Ethiopia [46–49]. This may be due to hormonal and genetic differences that promote slightly greater somatic growth in males. As a result, females are more likely to fall below the 2,500 g LBW threshold, even if they are appropriately grown for their gestational age [50].

This study also revealed that mothers who experienced pregnancy-related complications during their index pregnancy were at higher risk of delivering an LBW infant than mothers who didn’t experience pregnancy-related problems. This finding is consistent with studies conducted in Northern Ethiopia, Bale zone, Tigray, and Southeast Ethiopia [40, 51].

Our study found that mothers who smoked tobacco during pregnancy had a significantly higher risk of delivering low birth weight infants compared to non-smokers, consistent with findings from Bangladesh and Turkey [48, 49, 52, 53]. This association may be explained by the adverse effects of smoking on fetal growth, as maternal smoking is known to increase the risk of cognitive disability, preterm birth, and intrauterine growth restriction [54]. Nicotine causes vasoconstriction, which reduces oxygen flow to the fetus, while carbon monoxide forms carboxyhemoglobin, further impairing oxygen delivery to fetal tissues [55, 56]. Our finding of increased risk of LBW associated with lack of TT immunization in mothers is consistent with findings from an epidemiological study in rural Cameroon [57]. While TT vaccination primarily prevents neonatal and maternal tetanus, it may influence birth weight through several indirect pathways: (1) TT vaccination is often a marker of adequate antenatal care utilization, which itself is associated with better pregnancy outcomes including higher birth weight; (2) women who receive TT vaccination are more likely to receive other beneficial antenatal interventions (iron supplementation, nutritional counseling, infection screening); and (3) ANC and immunization contact provides opportunities for health education and early detection of pregnancy complications. It is also worth mentioning that, as a result of national initiatives to enhance maternal nutrition and birth outcomes, Bangladesh has recently switched routine iron-folic acid supplementation during pregnancy to multiple micronutrient supplementation [58].

This study has several strengths. It is a large, population-based prospective cohort in a developing country setting, which allowed precise measurement of birthweight and robust examination of associations between maternal, household, and health-seeking characteristics with the risk of LBW. All risk factors were measured before birth outcomes, reducing biases common in cross-sectional studies. We also used MUAC instead of BMI to assess maternal undernutrition; MUAC is a practical, low-cost alternative that does not require extensive training or calculations and avoids the problem of gestational weight gain masking true nutritional status.

The study also has several limitations. Infants with missing birthweight data or who died before the CHW’s first assessment were excluded, which may have introduced selection and survival biases and may have biased estimates toward underestimation of LBW. Although birthweight was measured within 72 h of delivery with most measurements obtained within the first 24 h, those recorded later may have been influenced by early postnatal weight loss, potentially leading to misclassification of LBW.

Some maternal variables, such as tobacco use and pregnancy complications, were self-reported and therefore subject to recall or social desirability bias. We also acknowledge that the data used in this study came from a rural site of East Bangladesh, which may limit its generalizability for the entire country. Additionally, although our literature review was comprehensive, analyses were constrained by the variables available in the dataset therefore some relevant variables, such as maternal infections and genetic factors, were not included, which may result in residual confounding.

Finally, we acknowledge that changes in socioeconomic and nutritional status, as well as healthcare utilization, may have occurred in the population since the study period, which was almost 15 years ago. For example, the prevalence of LBW in Bangladesh declined from 23% in the National LBW Survey (2015–16) to 14.8% in the MICS 2019, reflecting national progress. However, LBW prevalence remains higher in Sylhet division compared to national estimates according to BDHS 2017 and 2022 [59]. Nonetheless, the key risk factors identified here—maternal undernutrition, inadequate antenatal care, and low socioeconomic status—remain highly relevant, particularly among marginalized and underserved populations.

## Conclusion

This prospective cohort study demonstrates a high burden of low birthweight (LBW) in rural Bangladesh, driven by modifiable maternal, socioeconomic, and health system factors. These findings point to clear priorities for action.

Strengthening antenatal care (ANC) platforms is central. Programs should promote early and adequate ANC contacts and ensure delivery of a core package, including maternal nutrition support (e.g., multiple micronutrient supplementation), iron–folate use, tetanus toxoid immunization, and screening and management of pregnancy complications. Routine use of simple tools such as MUAC can help identify and target undernourished women.

Integrating tobacco cessation counseling into ANC and community platforms is also critical. In parallel, equity-focused strategies, such as community outreach, CHW-led follow-up, and support for women in poorer and remote households, are needed to improve access and continuity of care.

Together, these findings support an integrated, package-based approach to ANC strengthening to reduce LBW in Bangladesh and similar settings.

## Supplementary Information

Supplementary Material 1.

## Data Availability

Data and all the materials will be available from the corresponding author upon request.

## Abbreviations

ANC: Antenatal Care
aRR: Adjusted risk ratio
CI: Confidence Interval
LBW: Low birth weight
MNH: Maternal and newborn health. CHW: Community health workers
LMP: Last menstrual period
MUAC: Mid-upper arm circumference
IRB: Institutional review board
WHO: World health organization
