# Supplementary material for: Prevalence and risk factors of low birth weight in a rural district of Bangladesh: a prospective cohort study
Source: BMC Pediatr. 2026 May 12;26:623. doi: 10.1186/s12887-026-06960-x (PMC13339330; doi:10.1186/s12887-026-06960-x)
Supplement: Supplementary file 1 — Supplementary Material 1. [file 12887_2026_6960_MOESM1_ESM.docx]

**Supplementary table 1:** Risk factors of low birthweight using modified Poisson regression

| **Variables** | **RR (95% CI)** | **Adjusted RR (95% CI)** |
| --- | --- | --- |
| **Maternal characteristics** |  |  |
| **Age, years** |  |  |
| <20 | 1.36 (1.26, 1.48) | 1.43 (1.32, 1.56) |
| 20–24 | 1.21 (1.14, 1.29) | 1.23 (1.16, 1.32) |
| 25–29 | Ref | Ref |
| 30–34 | 0.96 (0.89, 1.04) | 0.92 (0.86, 1.00) |
| ≥35 | 1.07 (0.97, 1.17) | 0.98 (0.89, 1.08) |
| **Education** |  |  |
| None | 1.22 (1.15, 1.30) | 1.08 (1.00, 1.16) |
| Primary | 1.17 (1.11, 1.24) | 1.06 (1.00, 1.12) |
| Secondary or higher | Ref | Ref |
| **Mid-upper arm circumference, cm** |  |  |
| <22 | 1.26 (1.20, 1.33) | 1.19 (1.13, 1.25) |
| ≥22 | Ref | Ref |
| **Tobacco consumption during pregnancy** |  |  |
| No | Ref | Ref |
| Yes | 1.18 (1.11, 1.25) | 1.16 (1.09, 1.23) |
| **Passive smoking during pregnancy** |  |  |
| No | Ref | Ref |
| Yes | 1.14 (1.09, 1.20) | 1.06 (1.00, 1.11) |
| **Can go to health facility alone** |  |  |
| No | 1.14 (1.06, 1.22) | 1.11 (1.03, 1.19) |
| Yes | Ref | Ref |
| **Household characteristics** |  |  |
| **Household wealth quintiles** |  |  |
| 1 (poorest) | 1.60 (1.48, 1.73) | 1.45 (1.32, 1.59) |
| 2 | 1.53 (1.41, 1.66) | 1.39 (1.28, 1.52) |
| 3 | 1.33 (1.22, 1.45) | 1.24 (1.14, 1.35) |
| 4 | 1.17 (1.07, 1.28) | 1.11 (1.02, 1.22) |
| 5 (wealthiest) | Ref | Ref |
| **Pregnancy characteristics** |  |  |
| **Antenatal complication during pregnancy** |  |  |
| No | Ref | Ref |
| Yes | 1.09 (1.02, 1.16) | 1.12 (1.05, 1.19) |
| **Service factors** |  |  |
| **Antenatal care by a skilled healthcare provider** |  |  |
| No visit | 1.32 (1.23, 1.42) | 1.16 (1.07, 1.25) |
| 1–3 visits | 1.16 (1.08, 1.25) | 1.08 (1.00, 1.17) |
| ≥4 visits | Ref | Ref |
| **Taking iron tablet during pregnancy** |  |  |
| No | 1.16 (1.07, 1.25) | 1.10 (1.02, 1.19) |
| Yes | Ref | Ref |
| **Lifetime TT-Injection** |  |  |
| No dose | 1.24 (1.17, 1.32) | 1.17 (1.11, 1.25) |
| 1 dose | 1.34 (1.21, 1.48) | 1.26 (1.14, 1.39) |
| ≥2 doses | Ref | Ref |
| **Distance to health facility, km** |  |  |
| <15 | Ref | Ref |
| ≥15 | 1.08 (1.03, 1.13) | 1.11 (1.06, 1.17) |
| **Child factors** |  |  |
| **Sex of the infants** |  |  |
| Male | Ref | Ref |
| Female | 1.18 (1.13, 1.24) | 1.18 (1.13, 1.24) |

RR: Risk ratio, CI: Confidence Interval

**Supplementary table 2: Generalized Variance Inflation Factors (GVIF) for Multicollinearity Assessment**

| **Variables** | **GVIF** | **DF** | **Adjusted GVIF** |
| --- | --- | --- | --- |
| **Maternal characteristics** |  |  |  |
| Age, years | 1.26 | 4 | 1.03 |
| Education | 1.56 | 2 | 1.12 |
| Mid-upper arm circumference, cm | 1.02 | 1 | 1.01 |
| Tobacco consumption during pregnancy | 1.10 | 1 | 1.05 |
| Passive smoking during pregnancy | 1.07 | 1 | 1.04 |
| Can go to health facility alone | 1.07 | 1 | 1.03 |
| Household wealth quintiles | 1.46 | 4 | 1.05 |
| Complication during pregnancy | 1.02 | 1 | 1.01 |
| **Service factors** |  |  |  |
| Antenatal care by a skilled healthcare provider | 1.21 | 2 | 1.05 |
| Taking iron tablet during pregnancy | 1.02 | 1 | 1.01 |
| Lifetime TT-Injection | 1.06 | 2 | 1.01 |
| Distance to health facility, km | 1.06 | 1 | 1.03 |
| **Child factors** |  |  |  |
| Sex of the infants | 1.00 | 1 | 1.00 |
